# Feed Components and Timing to Improve the Feed Conversion Ratio for Sustainable Aquaculture Using Starch

**DOI:** 10.3390/ijms25147921

**Published:** 2024-07-19

**Authors:** Hideaki Shima, Taiga Asakura, Kenji Sakata, Masahiko Koiso, Jun Kikuchi

**Affiliations:** 1 RIKEN Center for Sustainable Resource Science, 1-7-22, Suehiro-cho, Tsurumi-ku, Yokohama 230-0045, Kanagawa, Japan; 2 Research Center for Subtropical Fisheries, Seikai National Fisheries Research Institute, Japan Fishery Research and Education Agency, 148 Fukaiota, Ishigaki 907-0451, Okinawa, Japan; 3 Graduate School of Medical Life Science, Yokohama City University, 1-7-29 Suehiro-cho, Tsurumi-ku, Yokohama 230-0045, Kanagawa, Japan; 4 Graduate School of Bioagricultural Sciences, Nagoya University, 1 Furo-cho, Chikusa-ku, Nagoya 464-8601, Aichi, Japan

**Keywords:** aquaculture, carbohydrates, data-driven approach, machine learning, nuclear magnetic resonance, stable isotope labeling

## Abstract

Aquaculture contributes to the sustainable development of food security, marine resource conservation, and economy. Shifting aquaculture feed from fish meal and oil to terrestrial plant derivatives may result in cost savings. However, many carnivorous fish cannot be sustained on plant-derived materials, necessitating the need for the identification of important factors for farmed fish growth and the identification of whether components derived from terrestrial plants can be used in feed. Herein, we focused on the carnivorous fish leopard coral grouper (*P. leopardus*) to identify the essential growth factors and clarify their intake timing from feeds. Furthermore, we evaluated the functionality of starch, which are easily produced by terrestrial plants. Results reveal that carbohydrates, which are not considered essential for carnivorous fish, can be introduced as a major part of an artificial diet. The development of artificial feed using starch offers the possibility of increasing the growth of carnivorous fish in aquaculture.

## 1. Introduction

Aquaculture is crucial for food supplies and is attracting attention as a source of protein and nutrition. Furthermore, as it affects the economy and prevents overharvesting, which is considered by some to be a major threat to marine resources; it can also play a role in mitigating poverty and is expected to contribute to multiple Sustainable Developing Goals (SDGs) [1,2,3,4]. However, aquaculture still faces challenges, such as poor feed efficiency, high cost, fish diseases, safety, quality, and environmental problems [2,4,5]. There is an urgent need to improve aquaculture using fish meal and oil, called “fish in fish out”. In other words, the effective use of terrestrial plants and other surpluses as feed must be optimized [6,7,8]. The feed conversion ratio (FCR) is an index of feed efficiency that indicates the amount of meat produced for a given unit of feed. Improvements in feeds are always required to obtain more meat. In aquaculture, the FCR varies greatly depending on the type of farmed fish. However, fish species that are less human-palatable, nutritional, or easy to eat have little value. Therefore, the FCR must be optimized for fish species to be worth farming.

*Plectropomus leopardus* (leopard coral grouper) is a commercially important carnivorous coral reef fish that inhabits temperate to tropical regions of the Western Pacific and Indian Oceans, from southern Japan to Australia [9,10]. *P. leopardus* is traded at high prices, particularly in Hong Kong [11]. Carnivorous fish, including *P. leopardus*, eat fish and crustaceans, whereas herbivorous fish eat aquatic plants and algae, and fish that eat both are omnivorous. These dietary habits affect their ability to use carbohydrates such as glucose [12].

Carbohydrates, including starch, store energy in terrestrial plants and can be easily and cost-effectively obtained due to their abundance. Additionally, reusing nitrogen and phosphorus while the plants are growing is possible in collaboration with microorganisms through composting and waste recycling, thereby affecting their environment [13]. Moreover, substituting carbohydrates with fish meal and oil can help preserve marine resources [2,14]. In aquaculture, carbohydrates are primarily used as glue in baits containing fish meat and oil, but their uses vary among fish species. When farming carnivorous fish, carbohydrates are used as binders in artificial feeds, with an emphasis on developing substitute proteins and lipids [12,15]. However, carbohydrates provide energy and prevent proteolysis, indirectly contributing to increased protein content [16,17], and they also help track metabolic pathways using stable carbon isotopes (^13^C) [18,19].

Nuclear magnetic resonance (NMR) spectroscopy helps us to understand the structure and motion of molecules, and it has been used for mixtures such as muscle, feces, water, and soil, followed by multivariate analyses [20,21,22,23]. Advances in computation, machine learning, and deep learning allow the analysis of big data and the extraction of important factors and environmental correlations [24,25,26,27].

In this study, to evaluate the function of feed components on growth during breeding, we farmed a large population of *P. leopardus* in a single tank; moreover, by sampling high-growth (Large) and low-growth (Small) individuals over time, we clarified the components in muscles important for growth in this species. In the farming protocol, we adopted a breeding method that allows *P. leopardus* to consume the appropriate type of feed according to its body length by providing sufficient nutrients in fortified feed suitable for each stage, from larva to adult, and then gradually reducing the type of feed based on the day after hatching. Finally, the fish were raised only with artificial bait.

The feed given at each stage and the sampled fish tissues were measured using NMR spectroscopy to understand their characteristics. Using machine learning methods, components in the muscles that characterize the size of *P. leopardus* were identified. Several characteristic factors were deduced, but there were two primary components, namely taurine, one of the fortified nutrients in feed, and glycine, a typical amino acid, which was not fortified and contributes to collagen formation. Furthermore, to identify substances in muscle important for size-related factors, we used machine learning methods and a Bayesian network for probabilistic causal inference. Surprisingly, our study indicated that even in *P. leopardus*, which is a carnivorous fish, providing sufficient glucose, a carbohydrate, and some small molecules are beneficial in the early stages of growth. Based on the identification of these important variables, we administered bait containing carbohydrates (starch) composed of ^13^C, a stable isotope, and attempted to visualize its metabolism. We found that starch can be used as a carbon source for glycine and other low-molecular-weight compounds. These results suggest a new way to improve the efficiency of aquaculture feed, which also contributes to the SDGs, by adjusting the timing of feeding carbohydrates (such as starch) which are abundantly obtained from terrestrial plants and some small molecules. Our results suggest that an improvement in the aquaculture strategy that is currently utilized is possible.

## 2. Results

### 2.1. Differences in Feed Components Based on NMR Spectroscopy Data

**Figure 1:**
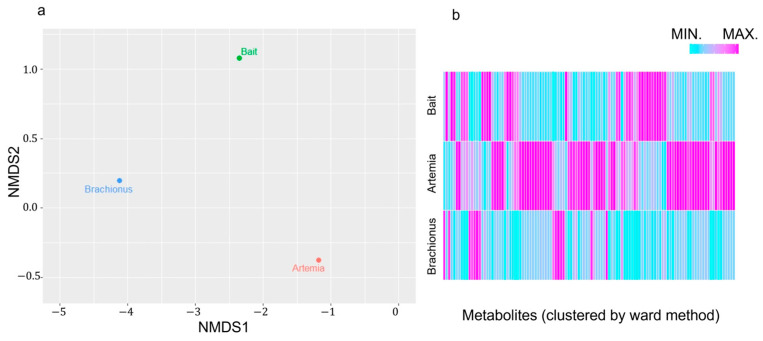
Differences based on feed type using distance information and the corresponding heatmap. (**a**) Nonmetric Multidimensional Scaling. Each circle shows distance data of feed similarity. Distance information was calculated as the Euclidean distance. (**b**) The ratio of signal intensities measured by nuclear magnetic resonance spectroscopy between feed types. Data are sorted by metabolite similarity (Ward method, arranged by similarity). A normalization step was performed for each metabolite.

We farmed *P. leopardus* in a single tank and fed them three types of food, brachionus, artemia, and artificial bait. Based on the facility’s experience, the food type ingested depends on the physical size of the oral cavity. Therefore, we assumed that the fish ate brachionus in the early stage of farming and artemia in the following stage. We measured the differences in the components of these feed types using NMR spectroscopy and calculated the Euclidean distance and the similarity of individual spectra (Figure 1). In Figure 1a, calculated distance data are shown on a Nonmetric Multidimensional Scaling map, indicating that each feed had different components. In Figure 1b, scaling and clustering were performed for each feed component to show the similarity between each feed type, indicating that each contained characteristic components.

### 2.2. Changes in Muscle Components during Growth Stages and Comparing Farmed and Wild-Captured Adult Fish

**Figure 2:**
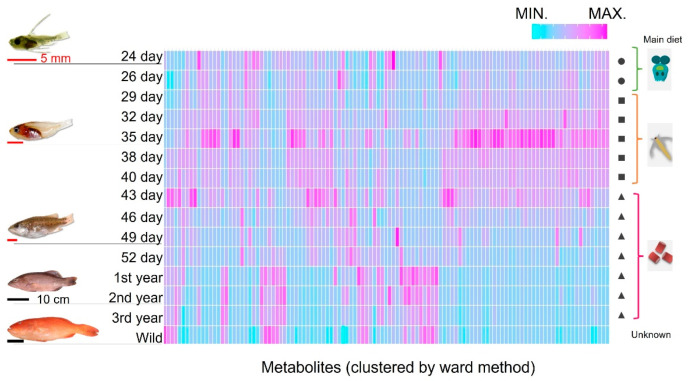
Heatmap of the components in the muscles of *P. leopardus* sorted by time (days) after hatching. Fish are arranged in descending order from top to bottom, with natural ocean fish at the bottom. Left, number of days since hatching; right, main feed type. Circles, squares, and triangles indicate brachionus, artemia, and artificial bait, respectively. The graph is sorted by metabolite similarity (Ward method, arranged by similarity). A normalization step was performed for each metabolite.

Next, we clustered the muscular components and visualized them in a heatmap to clarify how these components changed during *P. leopardus* growth. We also investigated whether these components were different from those in captured fish from the sea (Figure 2). However, as wild fish were obtained by fishing, the exact growth period was unknown. As obtaining juvenile fish is difficult, the average values for adult fish were used. Brachionus feed was removed from the tank after the 26th day of hatching, and artemia feed was removed after the 43rd day. Afterward, the *P. leopardus* were farmed only with artificial bait. Conversely, the diet of wild *P. leopardus* was unknown. Although the cells of heatmap were clustered by muscle components, the components differed by growth stage and the main diet. Additionally, the 1–3-year-old adult farmed fish and wild-captured fish clustered closer together in comparison the dispersion observed between juvenile clusters.

### 2.3. Extraction of Important Factors Determining Body Size in the Early Stages of Farming Using Machine Learning Methods

**Figure 3:**
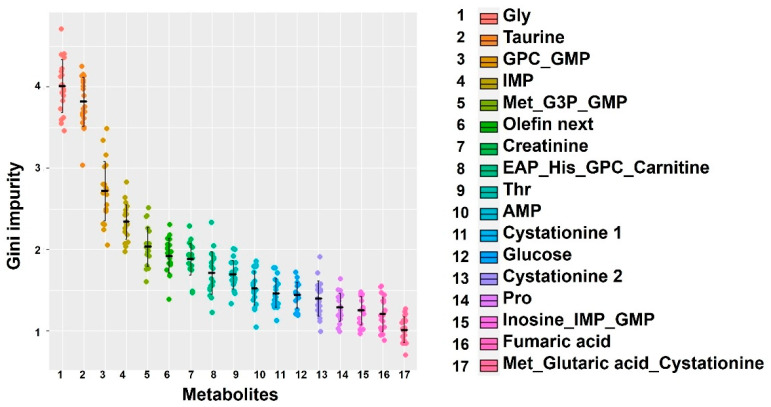
Distribution of common relevant factors to time fluctuations for separating high- and low-growth by machine learning. Relevant factors for classifying fish into high- and low-growth groups on each sampling day were calculated using the random forest method. It was repeated 20 times, and each Gini impurity was used as an importance score, with high implied importance. The x-axis represents the values on the right. In the list on the right, numbers next to names indicate multiple signals measured from the same component. Conversely, a signal with multiple names indicates overlapping signals. The horizontal black line indicates the average, and the vertical bar indicates one standard deviation.

Individual size variation emerged early. At the final sampling point, 52 days after hatching, mean length differed by 61% and mean weight differed by 350% between the high-growth and low-growth groups. A Shapiro–Wilk test for a Gaussian distribution did not deny the Gaussian distribution in the early days; however, the *p* value gradually decreased over time, reaching *p* = 0.0033 at 52 days, deviating from the Gaussian distribution. Under our farming conditions, each feed type had characteristic components, and the main feed also changed over time. Although the muscle components changed at each stage, we used machine learning methods to explore whether there were universally important factors affecting body size when classifying the high-growth and low-growth groups. We used a random forest approach, which is a machine learning method that uses decision trees for classification. Random forests are fast to calculate, even for big data, and they are highly accurate. However, this method is not suitable for unsupervised data. Since the Gini impurity, which is an index of importance calculated by the random forest, changes slightly with each calculation, we averaged 20 calculations (Figure 3). The figure shows components with an average Gini impurity of >1. Although many components had a value <3, there were two components that had a value close to 4: glycine and taurine.

### 2.4. Probabilistic Causal Inference Using Components That Explain Important Factors of Body Size Classification by Bayesian Networks

**Figure 4:**
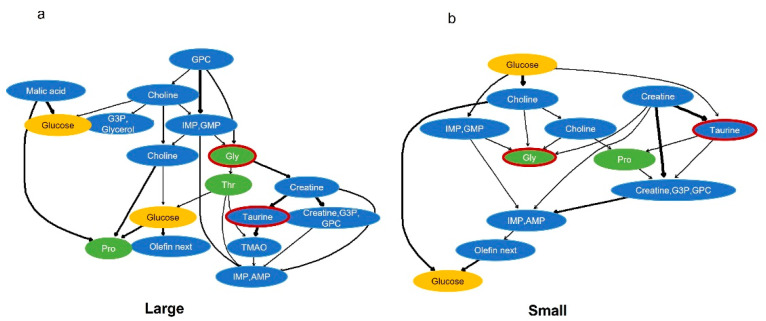
Probabilistic network for large and small fish based on important growth factors. Components that were used were extracted from random forest regression and from those that suggested a relationship between taurine and glycine. Part (**a**) was drawn using Large sample data (largest five individuals on each sampling day), and part (**b**) was drawn using Small sample data (smallest five individuals on each sampling day). The arrows indicate the probabilistic direction, and the line width indicates the strength of the connection. Red borders indicate important factors for growth (objective variables for regression), green indicates amino acids, yellow indicates glucose and blue indicates other components.

Subsequently, we performed Bayesian network analysis on the identified variables glycine and taurine (Figure 4) to infer probabilistic causal relationships. Bayesian networks can struggle with multiple conditions or weakly correlated variables. Thus, we analyzed the five biggest (Large) and five smallest (Small) individuals on each sampling day. Using random forest regression with glycine and taurine as objective variables for each of the large and small data, we identified key variables with high Gini impurity for each group. The reason for separately analyzing each size group was that this avoids noise from mixed group interactions. The results showed that both networks included glucose and the amino acids proline and threonine.

### 2.5. Analysis of Time-Series Changes in Important Factors for Body Size

**Figure 5:**
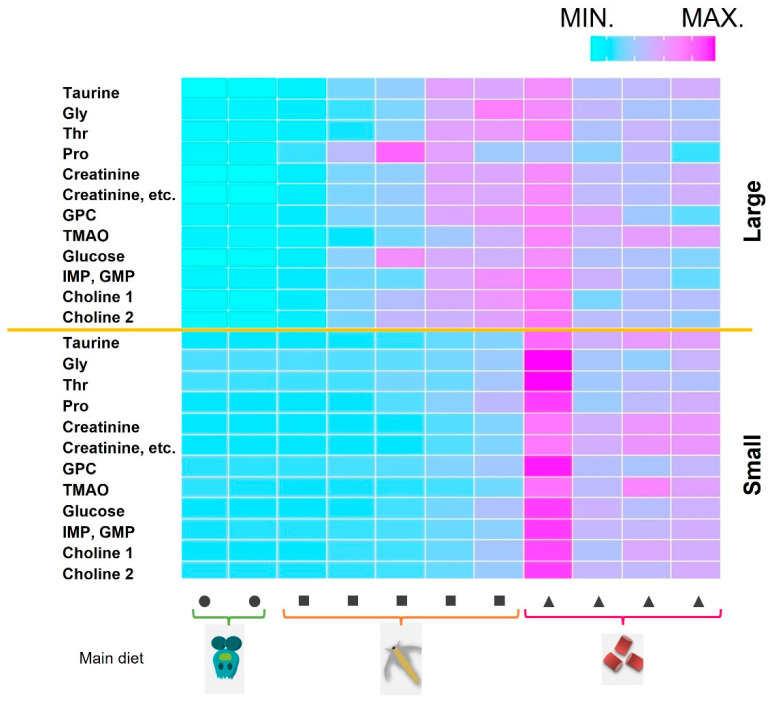
Time-series heatmap of growth-related components for fish groups with large and small body sizes. Growth-related components were extracted and used to depict the Bayesian network. Fish growth increases from left to right. The top half shows large fish, and the bottom shows the small fish. Scaling was performed in the time direction. The main feed type is shown at the bottom. Circles, squares, and triangles indicate brachionus, artemia, and artificial bait, respectively.

We analyzed time-series-scaled metabolic fluctuations to determine when important factors for growth are most effective during early farming (Figure 5). The heatmap was depicted with important elements for growth, such as taurine, glycine, threonine, proline, creatinine, glycerylphosphorylcholine (GPC), trimethylamine N-oxide (TMAO), glucose, inosine or guanosine monophosphate (IMP/GMP), and choline. These components were used in the random forest and Bayesian network mentioned above. The Small group had the highest value of all heatmap elements on the first day after switching to solely artificial bait, whereas the corresponding values of the Large group gradually increased after brachionus was removed. The highest amounts of proline and glucose were recorded when artemia was the main diet. Additionally, many components showed peak values at 1–2 sampling points before switching to artificial bait only, and these values were maintained. We also saw that there were no significant changes after the second sampling point, following the switch to artificial bait only.

### 2.6. Monitoring Starch Metabolism with ^13^C Labeling

**Figure 6:**
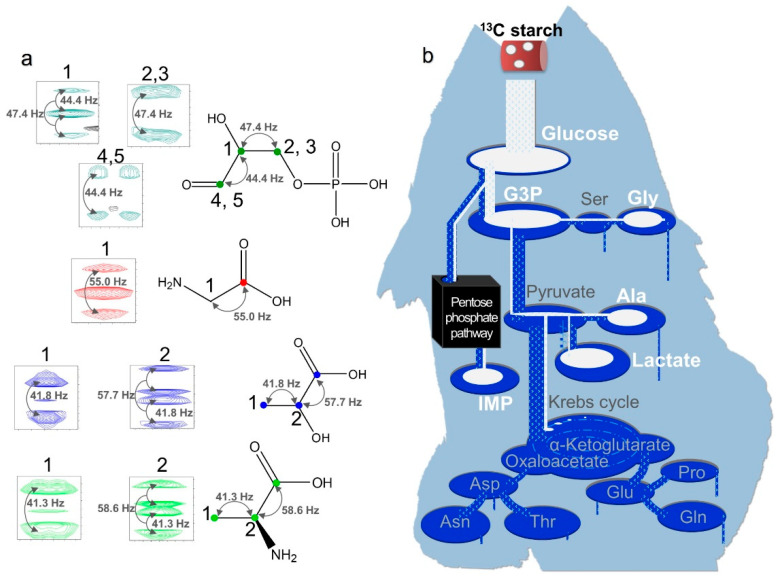
^1^H–^13^C HSQC spectra of water-soluble metabolites in *P. leopardus* muscle tissue. (**a**) Coupling constants between carbon atoms shown in Hz and indicated by arrows. Carbon atoms of the amino acids are successively numbered from the carboxyl carbon next to those carrying the amino group. Colored signals are ^13^C-labeled metabolites derived from ^13^C-labeled starch in the feed. Green, glyceraldehyde-3-phosphate (G3P); red, glycine; blue, lactate; light green, alanine. (**b**) Illustration of the metabolism of ^13^C-labeled starch into other compounds based on visual inspection of NMR data.

**Figure 7:**
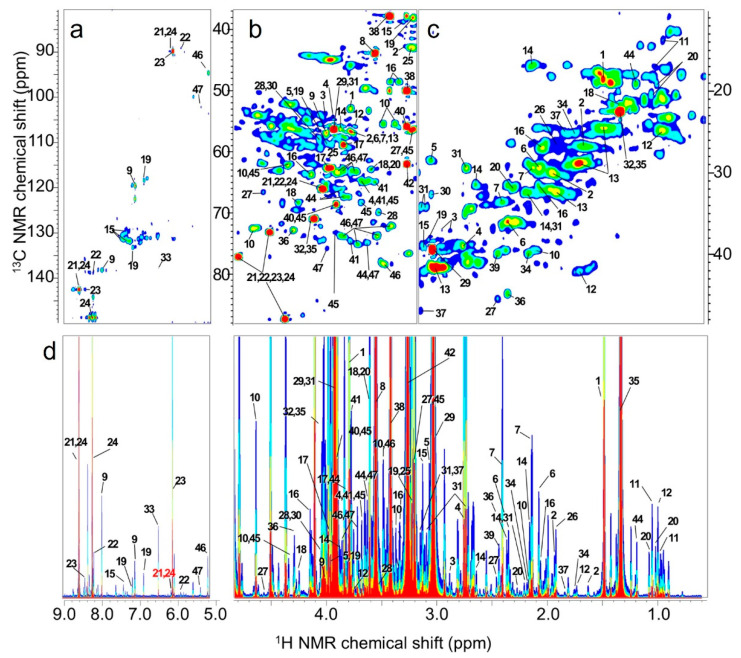
^1^H–^13^C correlation and ^1^H–^1^H *J*-resolved (2D-*J*res) NMR spectrum of muscle tissue. (**a**,**b**) Two-dimensional HSQC spectrum. (**c**) Two-dimensional 2D-*J*res spectrum. (**d**) Skyline plot of (**c**). The color indicates intensity. When blue is one, green is twice as strong, yellow is four times as strong, and red is eight times as strong (Supplementary Table S1).

The metabolism of ^13^C-labeled algal starch incorporated into *P. leopardus* muscle tissue with long-term (6 months) feeding was observed using heteronuclear single quantum coherence (HSQC) spectroscopy. The metabolites from ^13^C-labeled algal starch exhibited strong ^13^C–^13^C couplings and were easily distinguished from the other metabolites (Figure 6 and Figure 7). Our results confirmed that feed including ^13^C-labeled algal starch was digested into glucose and incorporated into muscle tissue (Figure 6). Surprisingly, some carbon in the fed starch was directly metabolized into glycine, while some was metabolized into inosinic acid monophosphate, glyceraldehyde-3-phosphate, alanine, and lactate.

## 3. Discussion

To clarify the relationship between the influence of feed on fish meat in aquaculture, we examined the components of feed and fish tissue over time (Figure 1 and Figure 2). In adult fish, although wild-caught fish and those that were farmed had distinctly different diets, the constituent components of their muscles were more similar than those during the changes occurring during early growth. Adult wild fish prey mainly on other fish [28], whereas the main ingredient of our artificial bait was fish meal. Conversely, some values were observed to have characteristic differences between wild and farmed fish. These included glucose (wild/farmed = 0.466), glycine (=0.788), and trimethylamine N-oxide (TMAO) (=5.472). Based on the stable isotope study (Figure 6), some of the glucose and glycine may be derived from carbohydrates such as starch. Additionally, seawater fish contain more TMAO than freshwater fish, and the compound has reported associations with carnivorous fish that mainly eat other fish [22,29,30,31]. In fact, the artificial bait we used contained up to 15% carbohydrates and 60% animal-derived ingredients, including fishmeal and shrimp meal. However, wild-caught fish may consume little carbohydrates. Compared to the stability of the constituent components of adult fish, the components extracted from the muscle of farmed fish may be significantly influenced by the feed type and timing of feeding.

In the early stages of farming, the muscle components differed depending on the developmental stage and the influence of their diet; however, at every sampling point, there were individuals that grew quickly and slowly. We investigated whether there were factors that universally influenced growth variation by random forest classification (Figure 3). The results clearly showed that two constituent factors were important, glycine and taurine. Glycine is present in large amounts in collagen, and it has been reported that, together with proline, glycine is important for collagen synthesis [32]. Proline also contributed to the classification of high-growth and low-growth fish, although to a lesser extent than glycine. Taurine is reported to be a critical nutrient for fish maturation, including *P. leopardus* [33,34,35]. Therefore, it is reasonable that glycine and taurine were identified as important factors separating the high-growth and low-growth groups using the random forest approach.

We set glycine and taurine as objective variables and performed random forest regression to identify components or metabolic networks related to glycine and taurine within each group. Furthermore, by conducting Bayesian network analysis using the extracted constituent factors, we estimated components that influence glycine and taurine (Figure 4). Bayesian network analysis suggested a relationship between glycine, threonine, and proline in the Large group, and a relationship between taurine and creatine in both groups. Furthermore, since two signals derived from glucose were embedded in the network, implying that the factors belonging to this module were equivalent to glucose, we inferred that the module was closely related to glucose utilization. Indeed, taurine is suggested to be related to glucose metabolism [36]. To investigate at which stage of early farming these factors were important, we created a heatmap in which each component was scaled over time (Figure 5). In the Small group, there was little fluctuation after reaching a peak the day we switched to artificial bait. Conversely, in the Large group, proline and glucose reached peaks when artemia was the main diet, and other components tended to be higher during that period. Thus, an intake of growth-related components such as glucose and proline when artemia was the main diet impacted later growth. The third sampling when artemia was the main diet was 35 days after hatching, and it was suggested that the transfer of glucose and required amino acids to the muscles before then might influence body size 43 days after hatching, when artificial bait was switched. This result is reasonable as proline and glycine are important for collagen synthesis [32].

Finally, to answer the question of how the carnivorous fish *P. leopardus* utilize glucose, we performed an experiment using artificial bait containing ^13^C stable isotopes incorporated into starch (Figure 6). The results revealed that after the starch was degraded, it was absorbed into glucose, and some of the carbon originating from the starch was directly metabolized into glycine. Carnivorous fish use glucose as an energy source, but the availability of glucose is low, and high doses of it leads to hyperglycemia [12,15,37,38]. However, our experiment using a stable carbon isotope showed that glucose was directly metabolized into amino acids that constitute collagen. However, no isotopes were found for proline or threonine, suggesting that some amino acids may be required when carbohydrates are ingested.

## 4. Materials and Methods

### 4.1. Ethics Statement

All experiments were conducted according to the principles and procedures approved by the guidelines for the care and use of live fish at the National Research Institute of Fisheries Science (Ishigaki, Japan) and RIKEN (Yokohama, Japan). As anesthetic chemicals such as 2-phenoxyethanol may influence metabolic profiling, all fish in our study were rapidly subjected to ice tightening—similar to other fishery and aquaculture products—during sampling. For wild fish, no specific permission was required at any sampling locations as fishing in public places is legal in Japan.

### 4.2. Fish Samples

To control the feed and environment, nearly all aquaculture samples were cultured at the Research Center for Subtropical Fisheries, Fishery Research and Education Organization (Ishigaki, Japan). The breeding conditions were as follows: On-shore aquaculture tank (20 ton) with use of sea water (Ishigaki, Okinawa-prefecture, Japan). Aeration was continuously performed. Brachionus, artemia, and artificial feed were used as feed. The artificial feed contained protein (>46.0%), fat (>10.0%), carbohydrates (<15.0%), fiber (<2.5%), calcium (>2.0%), and phosphorus (>1.0%) (Himezakura; Higashimaru, Kagoshima, Japan). The artificial bait comprised 60% animal-derived materials. Brachionus and artemia were cultured at the same institution as *P. leopardus*. Until 52 days of age, the body length of 96 individuals was measured every 3 days. The five largest individuals were considered the high-growth group (Large), and the five smallest individuals were the poor-growth group (Small). Wild samples were collected off the coast of Okinawa, Japan.

Three 18 cm, 1-year-old *P. leopardus* individuals not used in other experiments were cultured at RIKEN (Yokohama, Japan). To evaluate changes in dietary metabolism, carbon isotope labeling and fecal measurements were performed. For this experiment, feed was mixed with 20% *w*/*w* ^13^C-labeled algal starch (Cambridge Isotope Laboratories, Andover, MA, USA) and 80% *w*/*w* artificial feed (Himezakura). Feed was administered to *P. leopardus* for 6 months.

### 4.3. NMR

A total of 10 mg dried, powdered muscle tissue (or the total amount of muscle tissue if <10 mg) was extracted using 600 µL standardized potassium phosphate NMR buffer in deuterium oxide (^2^H > 90%) with 2, 2-dimethyl-2-silapentane-5-sulfonate (DSS) at 60 °C for 15 min and shaken for 15 min. After centrifugation at 25 °C for 5 min, the extracted supernatant was transferred into a 5 mm NMR tube (SHIGEMI, Hachiouji, Japan) for NMR measurements.

All 2D-Jres NMR spectra (magnitude-mode, gradient-enhanced, *J*-resolved with presaturation) were measured at 298 K on a Bruker Avance II DRU 700 NMR spectrometer. The measurement parameters of the 2D-*J*res were as follows: the time domain data size was 16,384 for F2 (^1^H) and 32 for F1 (*J*-coupling), the spectral width was 16 ppm for F2 and 50 Hz for F1, and the number of scans was 32. Following previous reports [39,40,41], 2D-*J*res spectroscopy was performed in a tilted manner, symmetrical to the horizontal axis through the F center. Skyline or sum-projection was performed as indicated. Additionally, *J*res projections were referenced and baseline-corrected. The obtained spectra were processed with TopSpin software version 4.0.6 (Bruker BioSpin, Rheinstetten, Germany) with a sine-bell window function, zero fillings to 128 points, tilt correction, and symmetrization. We selected the 2D-*J*res NMR projection due to a reduction in peak overlap as well as for its high sensitivity, which aids metabolite identification and data mining in spectral areas [39].

To assign the chemical shifts of metabolites, a 1-year-old fish was used and analyzed with ^1^H–^13^C HSQC using 320 scans with 256 data points for F1 (^13^C) and 1024 data points for F2 (^1^H) with spectral widths of 150 ppm for F1 and 14 ppm for F2. The HSQC spectra were processed using TopSpin software and annotated using SpinAssign [42]. For the ^13^C-labeling experiment, one fish cultured by labeled feeding underwent analysis with HSQC using 48 scans with 1300 data points for F1 (^13^C) and 1024 data points for F2 (^1^H) with spectral widths of 40 ppm for F1 and 14 ppm for F2. HSQC spectra were similarly processed and annotated. These extraction methods and parameters were based on previous studies [43,44,45].

### 4.4. Annotation and Normalization of NMR Spectra

Based on the position of the H–C correlation in the ^1^H–^13^C HSQC spectrum and the J-coupling constants from 2D-*J*res projection, 47 substances were detected in the water-soluble fraction of the metabolites from *P. leopardus* muscle tissue (Figure 7). These substances were assigned to distinguishable peaks in the 2D-*J*res projection spectrum. In subsequent analyses, intensity was taken as a composition ratio where necessary.

### 4.5. Analytics and Statistics

The 2D-*J*res spectra were processed into a data matrix using a peak-picking software based on the region of interest (ROI) using rNMR version 3.4.4 [46]. ROIs comprised information related to peak intensity and chemical shifts indicative of the region. ROIs that could not be annotated were excluded from the analyses. All calculations were performed using R software version 4.0.2 (http://www.R-project.org/, accessed on 1 June 2024) [47,48]. Additionally, we used the following packages: “vegan” for Nonmetric Multidimensional Scaling analysis, “bnlearn” for Bayesian network analysis, and “randomForest” for random forest analysis.

## 5. Conclusions

**Figure 8:**
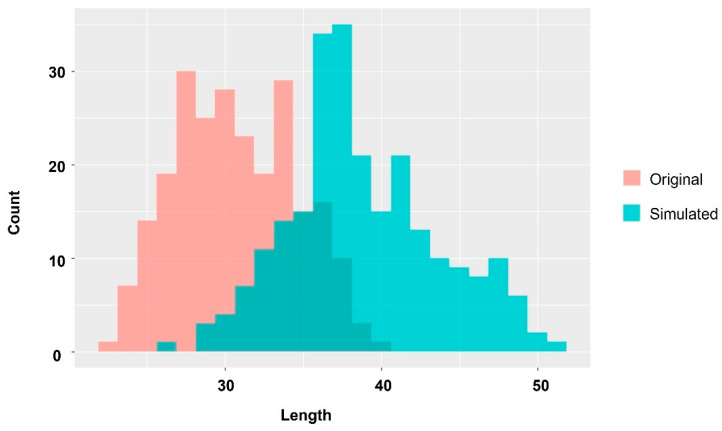
Histogram showing original growth distribution of *P. leopardus* and simulation results of aquaculture using starch effectively. Red histogram indicates length of *P. leopardus* at 52 days after hatching, and blue histogram indicates the simulated result. The simulation was scaled such that the average improvement was 30% and deviation was 10%.

In this study, we aimed to improve the FCR of farmed fish to improve food security. To clarify factors causing variation in body size during aquaculture, we farmed *P. leopardus* in a single tank and analyzed the components of their diets and muscle components measured with NMR spectroscopy and ^13^C stable isotope analysis, respectively, over time. The results revealed that *P. leopardus*, a carnivorous fish, could utilize carbohydrates and metabolize them into glycine, a characteristic component in the muscle of high-growth fish. We also identified amino acids that are considered essential for their effective use. At 52 days after hatching, body length of the Large group was approximately 60% larger than Small group, and the specimens in the Large group approximately 350% heavier than in the Small group. Based on these results, we conducted a simple simulation in which the Small group became average and the average group became slightly larger. This simple simulation estimated an improvement of about 25% in body length and about 100% in the FCR (Figure 8). This suggests that using cheap and abundant carbohydrates, which have not been previously given much attention in aquaculture, could improve artificial feed, increase aquaculture efficiency, and achieve multiple SDGs.

However, previous reports, including this study, have shown that other issues still remain when farming saltwater fish, such as osmotic pressure and temperature. Therefore, to avoid a food crisis, an improvement in the FCR in aquaculture must be considered not only when using starch from terrestrial plants but also for improving the aquaculture environment. Additionally, by applying our methods in other fields, the efficiency of agriculture and livestock farming can be improved.

## Data Availability

The raw data file comprising NMR signals is available at the following URL: http://dmar.riken.jp/NMRinformatics/ (accessed on 1 March 2024).

## Supplementary Materials

The supporting information can be downloaded at: https://www.mdpi.com/article/10.3390/ijms25147921/s1.
